# Premenopausal Syndrome and NAFLD: A New Approach Based on Gender Medicine

**DOI:** 10.3390/biomedicines10051184

**Published:** 2022-05-20

**Authors:** Livianna Carrieri, Alberto Ruben Osella, Fausto Ciccacci, Gianluigi Giannelli, Maria Principia Scavo

**Affiliations:** 1 Personalized Medicine Laboratory, National Institute of Gastroenterology IRCCS “S. de Bellis” Research Hospital, Via Turi 27, 70013 Castellana Grotte, Italy; livianna.carrieri@irccsdebellis.it; 2 Laboratory of Epidemiology and Biostatistics, National Institute of Gastroenterology IRCCS “S. de Bellis” Research Hospital, Via Turi 27, 70013 Castellana Grotte, Italy; arosella@irccsdebellis.it; 3 UniCamillus Saint Camillus International, University of Health Sciences, Via di Sant’Alessandro 8, 00131 Rome, Italy; fausto.ciccacci@unicamillus.org; 4 Scientific Direction, National Institute of Gastroenterology IRCCS “S. de Bellis” Research Hospital, Via Turi 27, 70013 Castellana Grotte, Italy; gianluigi.giannelli@irccsdebellis.it

**Keywords:** NAFLD, premenopausal syndrome, steatosis

## Abstract

Non-alcoholic fatty liver disease (NAFLD) is a multifactorial condition that affects 25% of the world’s population. There is a clear difference in both geographical distribution and sex in childbearing age. These differences are reduced when women become older and senescence begins. The factors that affect the likelihood of developing NAFLD in a premenopausal woman are an imbalance of sex hormones (especially in estradiol and androgen), microbiome dysregulation, insulin resistance, early menarche, the length of time that the woman breastfeeds for and polycystic ovarian syndrome (PCOS). The aim of this review is to identify various physical ailments that may not appear to be serious to young women but that then affect the onset of NAFLD in perimenopause and can degenerate into NASH. These conditions should also be considered in future clinical management, as well as in research opportunities, in order to customize the monitoring and treatment of NAFLD, considering gender medicine for those women who had early metabolic symptoms that were not considered to be significant at the time.

## 1. Introduction

Fat accumulation in the liver causes fatty liver disease as a result of hypertriglyceridemia, abnormal liver fat accumulation and the lack of alcohol consumption (the consumption of alcohol below 20 g/day is a factor that can be used to diagnose NAFLD). Non-alcoholic fatty liver disease (NAFLD) is a type of multifactorial syndrome, and 25% of the total population is affected by it [1]. The prevalence of NAFLD is high in all of the continents. The highest prevalence rates of NAFLD are reported in the Middle East (31%) and South America (32%), while the lowest are found in Africa (14% in 2016) [2]. In the United States and Europe, the prevalence rates of NAFLD are the same and constitute around 25% of the population, given the increase in wrong eating habits and sedentary lifestyles, similar to that observed in Asiatic populations [3]. Metabolically speaking, this mechanism is triggered by the consumption of fat and/or sugar in an unhealthy diet. As a result of this food choice, triglycerides accumulate in the blood, leading to obesity and heart disease [4]. These processes determine an alteration in the metabolic pathway of the liver, which leads to the activation of the oxidative response with the production of ROS at the level of mitochondria and peroxisome, with a consequential increase in very low density lipoproteins (VLDLs) in the blood stream [5]. The liver is unable to resolve inflammation foci, and for this reason, initial liver inflammation can easily evolve through interrupted tissue homeostasis, promoting hepatic steatosis, fibrosis and cirrhosis [6,7,8,9]. Furthermore, the incidence of obesity in the female population is not linked with cardiometabolic disease, such as NAFLD, at least not until menopause [10]. The differences between the liver of female and male mammals lies in the regulation of liver metabolism, which is under the control of sex hormones, particularly estrogen, and is subjugated to reproductive needs [11,12]. However, elevated androgen levels in transformed cells could have a tumor-promoting effect by stimulating cell growth in the liver [13]. Epidemiological data demonstrate that liver degeneration is more prevalent in men than in women, and hepatocellular carcinoma (HCC) development is not simply influenced by the levels of testosterone or estradiol but also depends on their ratio [14]. More is known about NAFLD in menopausal woman, while the knowledge on premenopausal women is dissonant and limited. Therefore, the incidence of NAFLD is higher in men than in women of reproductive age. With this perspective, the aim of this review was to evaluate the current state of NAFLD in premenopausal women when estrogenic decline has already begun. This could provide useful information to design lifestyle interventions, as well as to develop new therapeutical approaches on the basis of gender medicine to prevent and/or delay NAFLD.

## 2. Identification of Studies

**Figure 1:**
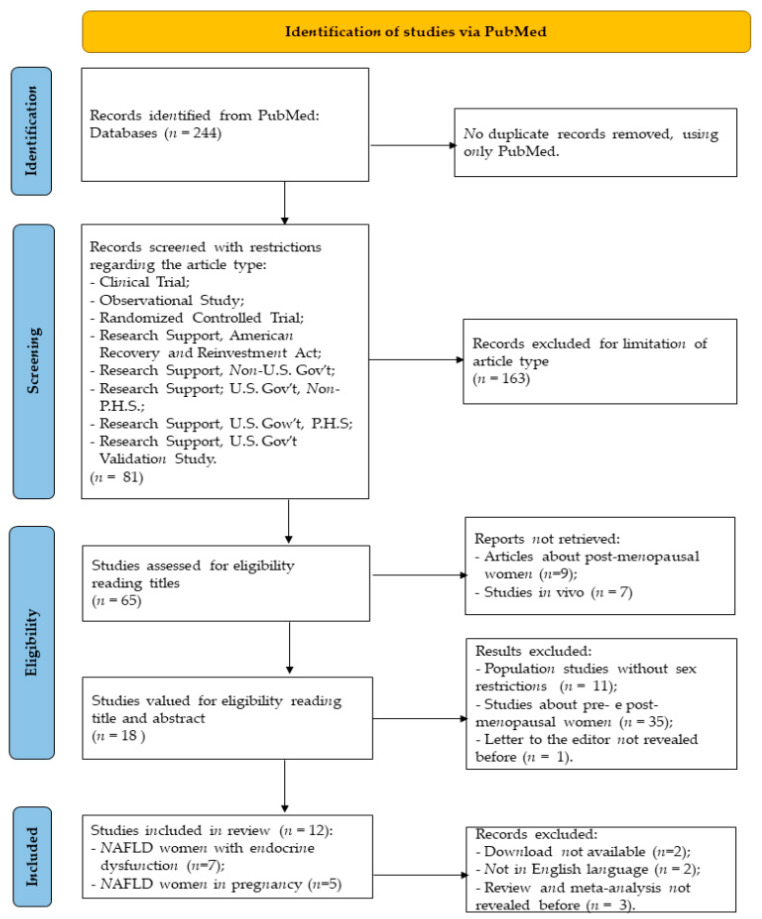
Identification of studies on PubMed. The methodology used to identify the work analyzed is summarized in 4 steps: identification, screening, eligibility and inclusion.

This review was conducted to investigate the epidemiology of NAFLD in premenopausal women, and the research was carried out on PubMed, using the following queries: ((Non alcoholic Fatty Liver Disease [Title]) OR (Nonalcoholic Fatty Liver Disease [Title]) OR (NAFLD [Title]) OR (Non-alcoholic Fatty Liver Disease [Title])) AND ((woman [Title]) OR (women [Title]) OR (female [Title]) OR (girl [Title])). A total of 244 articles were obtained, and they were published between 2006 and 2022. This review was carried out following the guidelines of the Preferred Reporting Items for Systematic Reviews and Meta-Analyses (PRISMA) 2020 statement [15]. The methods used for the research are described in the diagram in Figure 1.

After this in-depth study of the existing literature, the research results confirm the difficulty of diagnosing NAFLD in premenopausal women. The papers considered for this systematic study are reported in Table 1, which includes the age range of the patients, the pathogenesis, the year of publication and the bibliographic reference.

## 3. Hormones Involved in Development of NAFLD

**Figure 2:**
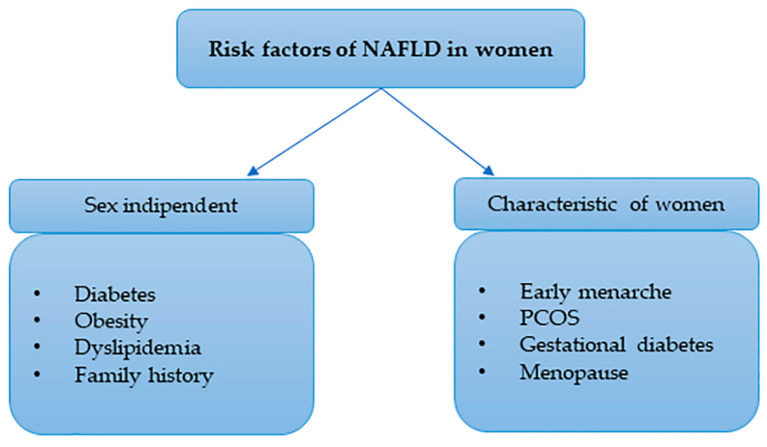
Risk factors in women. Risk factors in women may be gender-independent, such as diabetes, obesity, dyslipidemia and a family history of NAFLD, or they may be gender-dependent, particularly in relation to personal history, early menarche, PCOS, gestational diabetes and menopause.

Hamaguchi et al. underline the gradual age-related increase in NAFLD diagnosis in women (3.9% in the 21–39 age group; 7.6% in the 40–49 age group; 14.0% in the 50–59 age group; and 18.9% in the 60–69 age group) but not in men [28]. In a study conducted by Chung et al. [29], low HDL cholesterol (HDL-C), central obesity and homeostasis-model-assessment-estimated insulin resistance (HOMA-IR) were significantly associated with an increased risk of NAFLD in premenopausal women, while T2D, hypertriglyceridemia and central obesity were only significant risk factors in postmenopausal women, after adjustment for confounding variables. Sexual dimorphism in the development of NAFLD is due to a change in body composition and fat distribution, and it is due to some metabolic and hormonal changes that predispose the individual to the manifestation of this disease. These phenomena occur especially in women who are approaching menopause [30]. As can be seen in Figure 2, the factors that have been considered to explain the risk factors in woman (other than sexual dysmorphism) are sex independent: diabetes, obesity, dyslipidemia and a familiar history of NAFLD. Furthermore, some other factors relating to personal history also play a role: early menarche, polycystic ovarian syndrome (PCOS), gestational diabetes and menopause.

**Figure 3:**
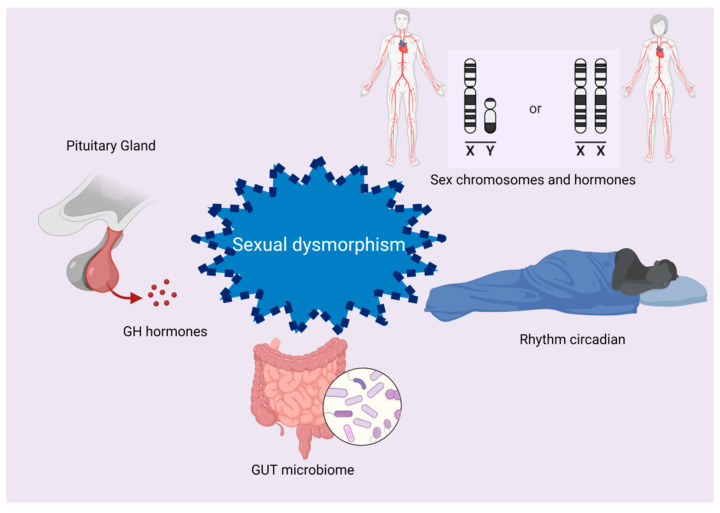
Sexual dysmorphism. Different factors contribute to sexual dysmorphism, including inactivation of chromosome X (in humans), complementary sex chromosomes, production of growth hormones (GHs), epigenetic mechanisms, intestinal microbiome, circadian rhythms (wake/sleep) and diet.

Furthermore, sexual dysmorphism is related to other factors, such as the inactivation of the X chromosome (in humans), complementary sex chromosomes, the production of growth hormones (GHs) and sex hormones, epigenetic mechanisms, the composition of the gut microbiome, the circadian rhythm and diet, as reported in Figure 3 [31]. NAFLD is thought to be triggered by the digestive-tract microbiota and its metabolites, such as short-chain fatty acids (SCFAs). In addition to a decreased amount of butyrate, the composition of the gut microbiota was found to be altered in NAFLD patients, but it can be rearranged by a low-glycemic-index Mediterranean diet combined with aerobic physical activity [32]. Liu et al. propose that the association between NAFLD and alterations in the gut microbiota and butyrate occurs during the premenopausal stage due to a low estrogen reduction [33,34,35].

The role of GHs is driven by sexual dimorphism, as it is produced by the pituitary gland, which is characterized by a pulsatile release in males; on the contrary, in infertile women, the release is continuous, and its function is to regulate the transcription of certain liver genes and protein production, including those related to lipid metabolism. GHs are responsible for the production of insulin-like growth factors (IGF-1) through autocrine and paracrine mechanisms [36]. An inadequate supply of GHs and IGF-1 can lead to depression, weight gain, changes in body composition and metabolic impairment [37]. Wang et al. determined a U-shaped relationship between GHs and age in the different genders and after sex stratification. The thresholds of age were 60 years in women and 50 years in men, with a strong regression showing that the GH level decreased in women aged <60 years (OR = 1.472, *p* < 0.001) and increased in men aged >50 years (OR = 0.711, *p* < 0.001) [38].

The GH axis may be protected by age in postmenopausal women by endogenous factors [39]. A growth spurt is caused by an increase in GHs and estrogen, followed by dramatic changes in physical development, leading to gender-specific body compositions [40]. Stimulated and spontaneous GH secretions are in fact higher in young women than in postmenopausal women and young men, with the difference being strongly correlated with circulating estradiol levels and the modulation of estrogen receptors [41,42]. Therefore, in comparison to healthy premenopausal women, men experience greater changes in HGH concentration. There is some evidence indicating that age-related decreases in growth hormones (GHs) are due to weakened hypothalamic feedback control [43]. The role of the balance of the alpha estrogen receptor (ER-α) and beta estrogen receptor (ER-β) is essential in the liver during lipid metabolism, acting as a regulator for the enzymes involved in the intake and release of cholesterol. An increased age-related decrease in the ratio of ER-α to ER-β in abdominal and femoral subcutaneous adipose tissue was observed in healthy pre- and post-menopausal women [44].

**Figure 4:**
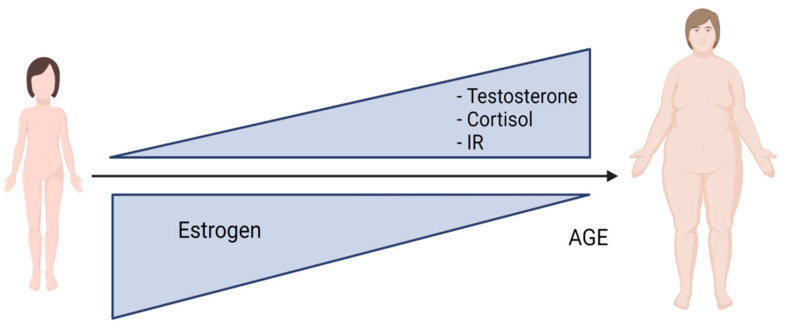
Modulation of hormones in premenopausal stage. During premenopausal stage, sex hormones, such as testosterone and estrogen, undergo an imbalance, together with cortisol, resulting in a dysfunction with a reduction in estrogen and increases in testosterone, cortisol and insulin resistance, resulting in increased body weight and abdominal fat.

All of this has an impact on the evolution of NAFLD because, although women physiologically produce high levels of estrogen at childbearing age (therefore maintaining high levels of receptors in the liver), the situation changes when estrogenic production decreases, leading women to have the same risk as men for metabolic diseases [45]. Another class of hormones involved in the evolution of NAFLD is androgens, including testosterone, dihydrotestosterone (DHT), dehydroepiandrosterone (DHEA), androsterone and androstenedione [46]. In childbearing age, men have a higher testosterone level (240–950 ng/dL) than women (8–60 ng/dL), so testosterone deficiency in humans has been associated with an increased accumulation of visceral fat and insulin resistance, and lower levels have been associated with obesity. In women, hyperandrogenism is linked to an increase in visceral fat and insulin resistance [47]. Furthermore, an increase in free testosterone is associated with prevalent NAFLD in adulthood, even in women without excess androgens. Visceral adiposity appears to play an important role in the relationship between testosterone and NAFLD in women. Testosterone may provide a potential novel target for NAFLD therapeutics, and future studies in premenopausal women should consider the importance of testosterone as a risk factor for NAFLD [48] because female androgen insufficiency (FAI) is most commonly caused by a decline in androgens during the premenopausal stage. However, natural menopause itself is not the cause of androgen deficiency in women [49]. In this regard, it has been observed that men and postmenopausal women are more likely to suffer from NAFLD than premenopausal women; this evidence suggests that the disparity could be due to sex steroid hormones (Figure 4). However, the mechanisms underlying the different risks of NAFLD in postmenopausal and premenopausal women still remain unclear [50]. Recently, a study reported that premenopausal women and women treated with synthetic hormones, such as oral contraceptives and HRT, both had an increased risk of lobular inflammation and hepatocyte injury, such as hepatocyte ballooning, Mallory–Denk body formation and lobular inflammation (*p* < 0.01), compared to men and postmenopausal women. At the same time, if the premenopausal women had an increased risk of liver injury, with an increase in the steatosis stage, they presented a lower stage of hepatic fibrosis than men and postmenopausal women. Despite their greater inflammation and injury to hepatocytes, premenopausal women also showed reduced levels of inflammatory markers [51]. Another study in the Japanese female population states that inflammatory markers are determinants of metabolic syndrome and weight gain in both pre- and post-menopausal women but that age is only a risk factor in premenopausal women [48]; in regard to this, during the menopausal transition, cortisol levels rise in some women. During the early stage of menopause or the premenopausal stage, the levels of cortisol in midlife and the menopausal transition slightly increase with aging. Overnight cortisol levels are associated with levels of FSH, testosterone, estrone, epinephrine and norepinephrine in urine. During the menopausal transition (MT), it would be helpful to examine daytime and nighttime patterns of cortisol to better understand the relationship between cortisol and vasomotor and sleep symptoms. A lack of correlation between social indicators and cortisol suggests that overnight cortisol levels are not related to stress, although they appear to correlate with various biological measures of stress, for example, epinephrine and norepinephrine. Future studies of stress may be better served by using dynamic measures, such as the cortisol awakening response [52]. NAFLD, which mimics hypercortisolism, is closely associated with obesity. Women are somewhat protected from the associated cardiometabolic effects of obesity, despite the prevalence being higher in them than in men. However, this protection wanes after menopause and during the premenopausal stage, supporting the hypothesis that estrogen plays a role in this process. The different metabolic responses to obesity observed in males and females may be a result of differences in glucocorticoid-metabolizing enzyme expression and activity between the sexes [53]. Another important role is played by insulin resistance (IR) in women with NAFLD. An interesting comparative study was carried out in 51 patients in the premenopausal stage, who had various grades of NAFLD and significant increases in fasting insulin and homeostasis model assessment (HOMA-IR) levels with advancing NAFLD grade, and control subjects. The conclusion reported a documented and combined interrelation between metabolic syndrome, IR and NAFLD in premenopausal women with metabolic syndrome, and showed that IR is correlated with NAFLD grade [54]. Recent studies have shown that, in adolescent women with obesity, early menarche is associated with both NAFLD and insulin resistance due to abdominal fat accumulation [55], which, during the fertile stage, could be associated with liver inflammation [56]. Therefore, in women that present early menarche, frequently, alanine aminotransferase, adult diabetes, higher triglyceride levels, BMI, waist circumference, higher C-reactive protein, cardiovascular morbidity and mortality, and advanced liver disease have been associated with NAFLD in the premenopausal stage [57].

## 4. PCOS Syndrome in Premenopausal Woman

PCOS is one of the most common endocrine disorders experienced by women during their reproductive years. The incidence is around 6–10% of the world female population [23]. There is an increased frequency of NAFLD in women with PCOS, which is one of the leading causes of chronic liver disease in the Western world [58]. Several studies demonstrate that estrogens play a regulatory role in NAFLD, which results in a higher prevalence in postmenopausal women than in premenopausal women and worsens after menopause [59]. Moreover, estradiol levels in women with PCOS are lower than those in women without PCOS, and the risk of these patients developing NAFLD is higher than that in premenopausal females without PCOS [60]. According to the meta-analysis conducted by Shengir and colleagues, premenopausal patients with PCOS have a 2.5-fold increased risk of NAFLD, and an increased BMI seems to be the major contributing factor [61]. PCOS is the cause of long, painful and abundant menstrual cycles. Cho IY demonstrated that women with long or irregular menstrual cycles and long premenopausal periods are at risk of developing NAFLD and should engage in healthy lifestyle behaviors, such as regular exercise and good eating habits [62,63]. Another consequence of PCOS is hirsutism, which is one of the first symptoms of this syndrome, along with oligomenorrhea. A study indicated that there is new evidence in the diagnosis of NAFLD to identify women at higher metabolic risk prior to referral for PCOS, thereby detecting those who may require further evaluation. In particular, this study carried out in women with hyperandrogenism, oligomenorrhea and female sexual dysfunction reports that an increase in the amount of sex-hormone-binding globulin (SHBG) is directly proportional to a pathological situation of NAFLD; in fact, an SHBG level of 33.4 nmol/L was recognized as the best threshold, with a sensitivity of 73.3% and a specificity of 70.7%, making this hormone a candidate for the monitoring of the dangerousness of having NAFLD in these types of patients, who may also not have metabolic syndrome [27].

## 5. Choline

**Figure 5:**
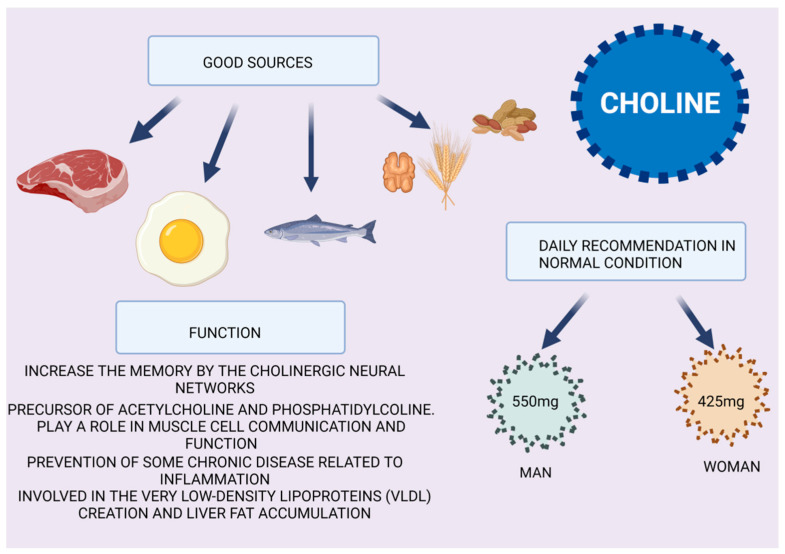
Choline properties. A variety of foods contain choline, with meat, fish, nuts, dairy products and eggs providing the richest sources. For adult men and women aged 19 years old or older, the adequate intake is 550 mg and 425 mg, respectively. Here, the principal functions of choline in cell metabolism and wellness are described.

NAFLD has been shown by a large number of studies to be caused by choline-deficient diets in humans and animals. Clearly, diet plays an important role in the prevention and development of NAFLD. In Figure 5, the foods that contain choline are described, along with the daily recommendations for men and women in normal conditions, and the major role of choline in health. It has been observed that, in diets poor in choline, NAFLD evolves rapidly and degenerates to NASH. In fact, a poor diet deficient in choline blocks the synthesis of phosphatidylcholine, which is essential for the creation of very low density lipoproteins (VLDLs), resulting in liver fat accumulation [64].

Several polymorphisms in genes involved in choline metabolism have been linked to liver damage susceptibility, including phosphatidylethanolamine N-methyltransferase and other enzymes [65]. Approximately 44% of premenopausal women will develop liver damage if they do not have enough choline; the remaining 56% of premenopausal women do not have these genetic polymorphisms that produce enough endogenous choline, and they also do not show signs of liver damage because, when estrogen levels increase, choline production also increases [66]. A higher choline requirement may be needed in patients who have a genetic variation that interferes with the normal metabolism of choline [67].

## 6. Pregnancy and Breast Feeding Are Alarm Bells for NAFLD in Premenopausal Women

It has been observed that pregnancy, despite being a physiological moment in a woman’s life, can evoke early symptoms of future pathologies such as NAFLD. Several studies, based on the correlation of events occurring in pregnancy and the manifestation of NAFLD over time, have shown an association between some conditions, such as gestational diabetes mellitus (GDM) or eclampsia, and NAFLD in older women [68]. This correlation is confirmed in the study conducted by Hagstrom et al., which considers a population of pregnant Swedish women diagnosed with NAFLD and a control population, including healthy women and women with PCOS [21]. Another study performed in 2020 by Liu and colleagues confirmed the correlation between eclampsia, GDM, pregnancy-associated hypertension (HTN) and NAFLD in the premenopausal stage. The markers of liver function and blood sugar were also altered similarly to those in NAFLD subjects [26]. Furthermore, high levels of selenoprotein *p* and adiponectin are likely to be responsible for linking hepatic steatosis to impaired glucose homeostasis during pregnancy and aging, especially in patients with NAFLD in premenopausal [69,70,71]. There is also evidence to suggest that parous women may suffer from an increased risk of NAFLD in later life (during the perimenopausal stage) when breastfeeding for a short period of time (ideally less than 6 months) while pregnant. Some cardiometabolic disorders may be linked to a short duration of breastfeeding, which could explain the connection [72]. Other recent studies have also correlated the increased incidence of NAFLD syndrome in premenopausal women with the period of breastfeeding after pregnancy. In 2019, Ajmera showed that the beneficial effects of breastfeeding at the metabolic level that persist over time (even after 25 years) were correlated with the manifestation of NAFLD in a population of about 844 women observed for 25 years after giving birth and breastfeeding. These women were divided according to the period of breastfeeding, and after 25 years, most premenopausal women with NAFLD were detected in the group that had breastfed for less than a month. In women who have breastfed for a longer period, lower values of BMI, HOMA-IR, triglycerides, total cholesterol (CHOL-T) and LDL (LDL-C) were found [24]. Park and colleagues published a cross-sectional study of 6893 Korean parous women aged 30–50 years from the Korean National Health and Nutrition Examination Survey, reporting that longer breastfeeding had a protective effect against NAFLD (as detected using the hepatic steatosis index) in the later life of parous women with ≥12 months’ duration of breastfeeding when compared to women with <1 month duration of breastfeeding [73]. Mantovani collected some of the work concerning breastfeeding and the prevention of NAFLD in adulthood, particularly during the premenopausal stage, and reinforced the assertion that breastfeeding contributes to the long-term health of the mother and protects against NAFLD [74].

## 7. Conclusions

As part of a systematic study of non-alcoholic fatty liver disease, we wanted to place an emphasis on the evolution of pathology in premenopausal women, as scientific evidence supports the hypothesis of long-term prevention, taking into account the childbearing life of patients. With the onset of the menopause in women’s lives, there is evidence that they are prone to more complications because estrogen levels physiologically decrease. However, evidence supports the hypothesis concerning the role of the change in the gut microbiota, which may cause estrogen reduction. Estrogens may also protect against insulin resistance, atherosclerosis and cardiovascular diseases due to their important role in liver fat accumulation. As a result of the reduction in estrogen production, predominant effects include an increase in food intake and a reduction in energy expenditure, but there is also the potential for metabolic effects on tissues such as the liver, for example, ectopic fat accumulation. In a recent review paper on NAFLD in middle-aged women, it was stated that there is no effective pharmacological treatment available, and lifestyle modifications, such as weight loss and exercise, are recommended first. In this review, we highlighted the conditions that can improve the manifestation of NAFLD in premenopausal woman, giving a new point of view from which to monitor patients, with the consideration of their life history. Having transient symptoms of metabolic imbalances at a young age, which apparently die down, can also be fertile ground for the development of pathologies such as NAFLD at premenopausal age.

**Figure 6:**
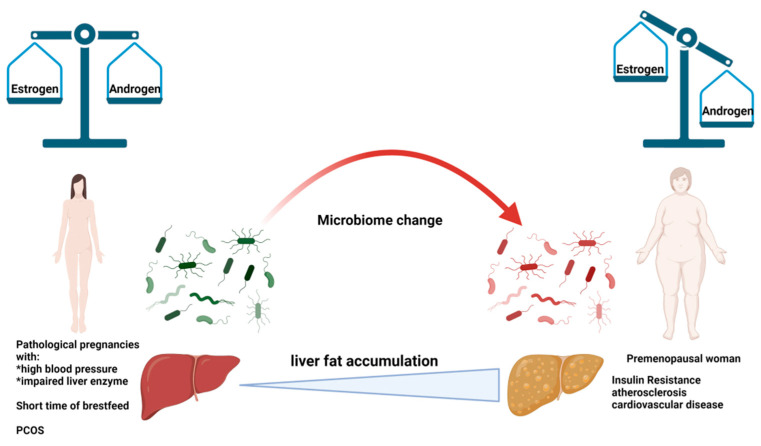
Causes that increase the risk of developing NAFLD at premenopausal age. Imbalance of sex hormones, changes in the intestinal microbiota, pathological pregnancies with increased blood pressure, pregnancies with diabetes, dysregulation of liver enzymes, short duration of breastfeeding and PCOS during the fertile age may increase the risk of being affected by premenopausal NAFLD.

In fact, it has already emerged that, in the detection of hormonal dysfunctions, such as sex hormone imbalances, pathological pregnancies with the development of a comorbidity (such as IR, high blood pressure, impaired liver enzymes, a short breastfeeding time and PCOS in young or older women) adversely affect susceptibility to NAFLD at an age very close to menopause (Figure 6). We are confident that accurate knowledge of the woman in adolescence, the promotion of healthy environmental habits, and pregnancy and breastfeeding management, as well as lifestyles that include a healthy diet and physical activity, could lead to a reduction in the frequency of NAFLD in women during their active age. These conditions should also be considered for future clinical management, as well as research opportunities, in order to customize monitoring and therapeutic approaches, considering gender medicine for women who had early metabolic symptoms that were not considered to be significant at the time.

## Figures and Tables

**Table 1:** List of the studies considered for the systematic study. These studies included patient age range and pathogenesis.

Age Group	Pathogenesis	Population Study	Study Duration	Reference		Year of Publication
Young	PCOS	200 women with PCOS	1999–2004	Setji, T. L. et al.	Nonalcoholic steatohepatitis and nonalcoholic fatty liver disease in young women with polycystic ovary syndrome. J. Clin. Endocrinol. Metab. [16]	2006
Fertile Adult	PCOS	41 consecutive non-pregnant women with PCOS and 31 non-pregnant heathy women	2005–2006	Cerda, C. et al.	Nonalcoholic fatty liver disease in women with polycystic ovary syndrome.J. Hepatol. [17]	2007
All range age	Hormones	223 women (110 with previous gestational diabetes and 113 without previous gestational diabetes)	2002–2010	Forbes, S. et al.	Increased prevalence of non- alcoholic fatty liver disease in European women with a history of gestational diabetes. Diabetology [18]	2011
Middle age and elderly	Problematic Pregnancy	5911 women with known reproductive histories	2010	Liu, Y. et al.	Association between history of abortion and nonalcoholic fatty liver disease in middle-aged and elderly Chinese women. Ann. Epidem [19]	2013
Pre-Menopausal	PCOS	110 women (71 with hepatosteatosis and 39 without hepatosteatosis)	2011–2014	Vassilatou, E. et al	Increased prevalence of polycystic ovary syndrome in premenopausal women with nonalcoholic fatty liver disease.Eur. J. Endocrinol. [20]	2015
Pre-Menopausal	Problematic Pregnancy	1960416 women divided into groups of non-NAFLD, non-PCOS and non-NAFLD with PCOS	1992–2011	Hagström, H. et al.	Adverse outcomes of pregnancy in women with non-alcoholic fatty liver disease. Liver int. [21]	2016
Pre-Menopausal	Obesity and PCOS	250 pre-menopausal women (132 with NAFLD and 158 non-NAFLD)	2007–2010	Vassilatou, E. et al.	Visceral adiposity index for the diagnosis of nonalcoholic fatty liver disease in premenopausal women with and without polycystic ovary syndrome. Maturitas [22]	2018
All range age	PCOS and Hormone imbalance	2706062 women divided into PCOS cohort and control group	2000–2016	Kumarendran, B. et al.	Polycystic ovary syndrome, androgen excess, and the risk of nonalcoholic fatty liver disease in women: A longitudinal study based on a United Kingdom primary care database. PLoS Med. [23]	2018
Pre-Menopausal	Hormone imbalance	844 women with reported lactation duration	1985–2010	Ajmera, V. et al.	Longer lactation duration is associated with decreased prevalence of non-alcoholic fatty liver disease in women. J.Hepatol. [24]	2019
Pre-Menopausal	Hormone imbalance	210 pre-menopausal women with confirmed NAFLD	2020	Sarkar, M. A. et al.	Testosterone is Associated With Nonalcoholic Steatohepatitis and Fibrosis in Premenopausal Women With NAFLD.Clin. Gastroenterol. Hepatol. [25]	2021
Pre-Menopausal	Problematic Pregnancy	877 pregnant women	2014–2017	Jung, Y. M. et al.	The risk of pregnancy-associated hypertension in women with nonalcoholic fatty liver disease. Liver Int. [26]	2020
All range age	Hormones imbalace and sexual Dysfunction	66 women with oligomenorrhea and/or hyperandrogenism and 233 women with sexual dysfunction	2020	Di Stasi, V. et al.	SHBG as a Marker of NAFLD and Metabolic Impairments in Women Referred for Oligomenorrhea and/or Hirsutism and in Women With Sexual Dysfunction. Front. Endocrinol. (Lausanne) [27]	2021

## Data Availability

Not applicable.
